# Supplementary material for: Player load in male elite soccer: Comparisons of patterns between matches and positions
Source: PLoS One. 2020 Sep 21;15(9):e0239162. doi: 10.1371/journal.pone.0239162 (PMC7505455; doi:10.1371/journal.pone.0239162)
Supplement: S1 Table — CD: central defender; ED: external defender; CM: central midfielder; EM: external midfielder; ATT: attacker. For p-values, bold text indicates significance at α = .05. (DOCX) [file pone.0239162.s001.docx]

**S1 Table. Cross-correlations [95% CI] of mean position values (z-scores) across all matches (*n* = 34) at zero lag for accelerations, decelerations, sprint distance, and high-intensity running distance.**

CD: central defender; ED: external defender; CM: central midfielder; EM: external midfielder; ATT: attacker.

For p-values, bold text indicates significance at α = .05.

|  | **CD** | **ED** | **CM** | **EM** | **ATT** |
| --- | --- | --- | --- | --- | --- |
| *Accelerations* | | | | | |
| **CD** | --- |  |  |  |  |
| **ED** | 0.63 **(p<.001)**  [0.48, 0.75] | --- |  |  |  |
| **CM** | 0.55 (**p<.001**)  [0.37, 0.68] | 0.54 (**p<.001**)  [0.36, 0.68] | --- |  |  |
| **EM** | 0.32 (**p=.005**)  [0.11, 0.50] | 0.54 (**p<.001**)  [0.37, 0.68] | 0.40 (**p=.001**)  [0.20, 0.57] | --- |  |
| **ATT** | 0.41 (**p<.001**)  [0.21, 0.58] | 0.64 (**p<.001**)  [0.49, 0.75] | 0.45 (**p<.001**)  [0.26, 0.61] | 0.45 (**p<.001**)  [0.26, 0.61] | --- |
| *Decelerations* | | | | | |
| **CD** | --- |  |  |  |  |
| **ED** | 0.65 **(p<.001)**  [0.51, 0.76] | --- |  |  |  |
| **CM** | 0.39 **(p<.001)**  [0.19, 0.56] | 0.45 **(p<.001)**  [0.25, 0.60] | --- |  |  |
| **EM** | 0.36 **(p<.001)**  [0.15, 0.54] | 0.50 **(p<.001)**  [0.31, 0.64] | 0.18 (p=.098)  [-0.03, 0.39] | --- |  |
| **ATT** | 0.38 **(p<.001)**  [0.18, 0.55] | 0.51 **(p<.001)**  [0.33, 0.65] | 0.25 **(p<.023)**  [0.04, 0.44] | 0.54 **(p<.001)**  [0.36, 0.68] | --- |
| *Sprint distance* | | | | | |
| **CD** | --- |  |  |  |  |
| **ED** | 0.17 (p=.132)  [-0.05, 0.37] | --- |  |  |  |
| **CM** | 0.09 (p=.399)  [-0.13, 0.31] | 0.33 **(p=.002)**  [0.13, 0.51] | --- |  |  |
| **EM** | -0.11 (p=.311)  [-0.32, 0.11] | 0.12 (p=.290)  [-0.10, 0.33] | 0.13 (p=.254)  [-0.09, 0.34] | --- |  |
| **ATT** | 0.02 (p=.838)  [-0.20, 0.24] | 0.02 (p=.875)  [-0.20, 0.23] | 0.38 (**p<.001**)  [0.17, 0.55] | 0.32 (**p=.004**)  [0.11, 0.50] | --- |
| *High-intensity running distance* | | | | | |
| **CD** | --- |  |  |  |  |
| **ED** | 0.08 (p=.487)  [-0.14, 0.29] | --- |  |  |  |
| **CM** | -0.15 (p=.185)  [-0.35, 0.07] | 0.52 (**p<.001**)  [0.34, 0.66] | --- |  |  |
| **EM** | 0.03 (p=.797)  [-0.19, 0.24] | 0.36 (**p<.001**)  [0.15, 0.53] | 0.11 (p=.347)  [-0.11, 0.32] | --- |  |
| **ATT** | 0.02 (p=.834)  [-0.19, 0.24] | -0.03 (p=.811)  [-0.24, 0.19] | 0.01 (p=.921)  [-0.21, 0.23] | 0.48 (**p<.001**)  [0.30, 0.63] | --- |
