# Supplementary material for: Player load in male elite soccer: Comparisons of patterns between matches and positions
Source: PLoS One. 2020 Sep 21;15(9):e0239162. doi: 10.1371/journal.pone.0239162 (PMC7505455; doi:10.1371/journal.pone.0239162)
Supplement: S2 Table — CD: central defender; ED: external defender; CM: central midfielder; EM: external midfielder; ATT: attacker. A negative lag means that the first time series (player position in table columns) shifts to the left relative to the second time series (player position in table rows). (DOCX) [file pone.0239162.s002.docx]

**S2 Table. Maximum cross-correlations (corresponding lag) of mean position values (z‑scores) across all matches (*n* = 34) for accelerations, decelerations, sprint distance, and high-intensity running distance.**

CD: central defender; ED: external defender; CM: central midfielder; EM: external midfielder; ATT: attacker.

A negative lag means that the first time series (player position in table columns) shifts to the left relative to the second time series (player position in table rows).

|  | **CD** | **ED** | **CM** | **EM** | **ATT** |
| --- | --- | --- | --- | --- | --- |
| *Accelerations* | | | | | |
| **CD** | --- |  |  |  |  |
| **ED** | 0.63 (0) | --- |  |  |  |
| **CM** | 0.55 (0) | 0.54 (0) | --- |  |  |
| **EM** | -0.56 (-27) | 0.54 (0) | 0.40 (0) | --- |  |
| **ATT** | 0.41 (0) | 0.64 (0) | 0.45 (0) | 0.45 (0) | --- |
| *Decelerations* | | | | | |
| **CD** | --- |  |  |  |  |
| **ED** | 0.65 (0) | --- |  |  |  |
| **CM** | 0.39 (0) | 0.45 (0) | --- |  |  |
| **EM** | 0.36 (44) | 0.50 (0) | 0.44 (30) | --- |  |
| **ATT** | 0.39 (-4) | 0.51 (0) | -0.32 (21) | 0.54 (0) | --- |
| *Sprint distance* | | | | | |
| **CD** | --- |  |  |  |  |
| **ED** | -0.39 (54) | --- |  |  |  |
| **CM** | -0.37 (33) | 0.37 (8) | --- |  |  |
| **EM** | 0.36 (11) | -0.29 (-29) | -0.37 (-28) | --- |  |
| **ATT** | 0.53 (6) | 0.46 (11) | 0.38 (0) | 0.32 (0) | --- |
| *High-intensity running distance* | | | | | |
| **CD** | --- |  |  |  |  |
| **ED** | 0.36 (23) | --- |  |  |  |
| **CM** | 0.37 (-11) | 0.52 (0) | --- |  |  |
| **EM** | -0.39 (11) | -0.39 (-13) | -0.44 (-28) | --- |  |
| **ATT** | -0.32 (10) | -0.42 (-13) | -0.36 (32) | 0.48 (0) | --- |
