# Supplementary material for: Player load in male elite soccer: Comparisons of patterns between matches and positions
Source: PLoS One. 2020 Sep 21;15(9):e0239162. doi: 10.1371/journal.pone.0239162 (PMC7505455; doi:10.1371/journal.pone.0239162)
Supplement: Attachment — Submitted filename: Response to reviewers, PlosOne, R2.docx [file pone.0239162.s005.docx]

Dear Editor:

We thank you and the reviewers for helping us get our paper better and do hope you find the present version of the manuscript acceptable for publication.

**Comment 1,** Reviewer #2: The paper "Player load in male elite soccer: comparisons of patterns between matches and positions” is a good contribution for the current state of the art in this specific topic.
Previously, I recommended to change your title and you in fact provide a changed title. However, I understand your answer to this topic, I still does not share the same idea because I really consider that player load and the other variables as well are equally relevant for the study. But I will let to your consideration.

**Response 1:** Thank you for accepting our view on this, we appreciate the understanding.

**Comment 2,** Reviewer #2: Thus, I would like to suggest to change the first line sentence of the abstract to “Our primary aim was to explore the development of player load throughout match time (i.e., the pattern) using moving 5-min windows in an elite soccer team and our secondary aim was to compare player load patterns between different positions within the same team.”

**Response 2:** We agree, and have made the change you request. The missing “was” was initially removed as a grammatical move to reduce words where we could, since we added more results to the abstract. To remain under the 300 word limit, we have now changed “which was also present in the majority of individual matches” to “which also occurred in the majority of individual matches”.
